# Dysfunction of GABAergic interneurons underlies altered neural network oscillations associated with epileptiform activity in PPT1-deficient mice

**DOI:** 10.1038/s41398-026-03843-8

**Published:** 2026-02-02

**Authors:** Jia Tong, Weizhen Liu, Qianqian Wang, Huifang Yang, Ziyan Gao, Wanliu Wu, Jie Liu, Wenqiang Li, Chengbiao Lu

**Affiliations:** 1 The Second Affiliated Hospital of Xinxiang Medical University, He’nan Key Laboratory of Biological Psychiatry (Xinxiang Medical University), Xinxiang, He’nan 453002 China; 2 Institute of Psychiatry and Neuroscience, Xinxiang Medical University, Xinxiang, He’nan 453003 China; 3 He’nan Medical Key Laboratory for Research of Trauma and Orthopedics, The Third Affiliated Hospital of Xinxiang Medical University, Xinxiang, He’nan 453003 China; 4 He’nan Key Lab of Biological Psychiatry, He’nan Collaborative Innovation Center of Prevention and Treatment of Mental Disorder, Xinxiang Medical University, Xinxiang, He’nan China; 5 He’nan International Joint Laboratory for Non-invasive Neural Modulation, Xinxiang Medical University, Xinxiang, He’nan 453003 China

**Keywords:** Molecular neuroscience, Psychiatric disorders, Physiology, Clinical genetics

## Abstract

The neuronal ceroid lipofuscinosis family of lysosomal storage diseases, also called *CLN1* disease, is characterized by the deficiency of palmitoyl-protein thioesterase 1 (PPT1). In this study, we investigated the impact of PPT1 deficiency on hippocampal GABAergic interneurons (INs) and associated neural network oscillations in a PPT1-KI (*CLN1* c.451 C > T (p.R151X)) mouse model. Using a combination of in vivo electrophysiology, immunostaining, and fiber photometry, we observed that PPT1 deficiency led to the activation of caspase 3 in parvalbumin-positive (PV^+^) INs, an increased activity of pyramidal neurons and theta/gamma oscillation power, and the disruption of theta-gamma cross-frequency coupling (CFC) in the early stage of the *CLN1* disease model. In the late stage of the *CLN1* disease model, we observed the reduced neuronal activity, extensive neuronal loss including PV^+^ INs, and the emergence of spontaneous epileptiform discharges and the pathological ripples. Treatment with diazepam partially restored oscillatory coupling and reduced seizure-like activities. Our research indicated that PPT1 deficiency leads to early selective impairment of PV^+^ INs, triggering overactivation of pyramidal neurons and network dysfunction, which consequently results in seizures and neurodegeneration. This research provides novel insights into the pathogenesis of *CLN1* disease and potential therapeutic strategies for the intervention of *CLN1* disease by improving the function of inhibitory INs via caspase inhibition.

## Introduction

Palmitoylation is a post-translational modification (PTM) where a palmitoyl group (C16:0) is covalently linked to the thiol group of cysteine residues at the C-terminus of a protein through a thioester bond [1]. Comprehensive and objective proteomic analyses of the mammalian brain have revealed over 600 palmitoylated proteins, with a significant proportion residing in synaptic regions [2–4]. These studies underscore the pivotal role of palmitoylation in synaptic neurotransmission.

PPT1, encoded by the *CLN1* gene [5–8], is a lysosomal enzyme [7] that mediates depalmitoylation by removing palmitate residues from S-acylated proteins [5, 9]. Mutations in the *CLN1* gene lead to PPT1 deficiency, which causes the most severe form of lysosomal storage diseases. Children with *CLN1* disease appear normal at birth but later experience retinal degeneration, leading to blindness, as well as impairments in learning, memory, and an increased risk of seizures [10–14].

PPT1 plays critical roles in neuronal transmission, deficiency of PPT1 causes abnormal and persistent membrane accumulation of the synaptic vesicle proteins, such as vesicle-associated membrane protein 2 and syntaxin 1 [15]. Recent study indicates that PPT1 knockout mice showed impairment of long-term potentiation (LTP) in the hippocampus in response to tetanic stimulation [16]. Nevertheless, the pathogenesis of epilepsy, a hallmark of *CLN1* disease within the nervous system, remains poorly understood. Consequently, examining the underlying mechanisms of PPT1 in modulating neural circuitry activity in vivo becomes especially crucial.

Despite a smaller in proportion (15% ~ 35%) [17], INs such as PV^+^ [18, 19] and SST^+^, can regulate the frequency of neural oscillations in the hippocampal region. By precisely controlling the frequency and phase of these oscillations, INs help synchronize and coordinate the activity of neural networks, which is crucial for memory encoding and retrieval. INs optimize the transmission and processing of information by regulating the activity of excitatory neurons, thereby achieving synchronization or desynchronization between different neuronal populations.

By using in vivo electrophysiological recordings, combined with histological staining and fiber photometry recordings, this study focuses on the effects of PPT1 deficiency on hippocampal INs, the activity of pyramidal neurons and neural circuit oscillations during the early and late stages of *CLN1* disease to uncover the cellular and neural network mechanisms. The findings offer new perspectives on understanding the pathogenesis of *CLN1* disease and suggest potential new therapeutic strategies for its treatment.

## Material and methods

### PPT1-KI (CLN1 c.451 C > T (p.R151X)) mutant mice

The strategy for point mutation of the *CLN1* gene to generate PPT-KI mice has been described in Fig. S1. In previous studies, PPT1-KI mice carrying c.451 C > T/c.451 C > T mutations are deficient in PPT1-protein as well as PPT1 enzyme activity [20]. C57BL/6 N mice were purchased from Beijing Vital River Laboratory Animal Technology Co., Ltd, Beijing, China (animal licence number:2016-0006) and used as the wild-type (WT) controls. All animals were housed and maintained in the specific pathogen-free animal facility of the Animal Experiment Center of the Institute of Psychiatry and Neuroscience of Xinxiang Medical University (XXMU) under a 12-h light/dark cycle. Animals had ad libitum access to food and water, except during food and water deprivation periods. All efforts were made to minimize animal suffering and reduce the number of animals used. Diazepam (3 mg/kg in 10% DMSO/saline, i.p.) [21] or vehicle (10% DMSO/saline) was administered to PPT1-KI epileptic mice, followed by electrophysiological recordings. Animal experiments were performed according to the regulations and requirements of the XXMU Animal Ethics Committee (No. XYLL2021053).

### In vivo electrophysiological recording

#### Stereotaxic surgery and electrode implantation

Mice were anaesthetized with an intraperitoneal injection of 1% pentobarbital sodium (0.45 ~ 0.5 mL/100 g). Anesthesia depth was confirmed by the absence of pedal reflexes. Anesthesia depth was monitored every 15 min, and supplemental doses (not exceeding 1/5 of the total initial dose) were administered if pedal reflexes reappeared. The mice were then secured in a stereotaxic frame with ear bars, and body temperature was maintained at 37 °C using a heating pad. The head was shaved with a razor, and a midline 5-mm incision was made using a sterile scalpel. The subcutaneous tissue was removed from the skull, and a cranium (~1.5 × 0.5 mm) was drilled (AP: 1.82 mm, ML:1.25 mm, DV:1.5 mm, corresponding to the CA1 region). Two steel screws are anchored to the anterior and posterior edges of the surgical site to secure the implant. After the endocranium was removed, a 4 × 2 micro wire electrode (KD-MWA-8, 25 μm nitinol wire, Kedou (Suzhou) Brain-Computer Technology Co., Ltd.) with 3 μm polyethylene glycol coating was implanted into the pyramidal cell layer of CA1. The craniotomy site was sealed with a sterile silicone elastomer (Kwik-Sil WPI) to prevent brain injury. After surgery, the implanted electrodes and screws were cemented integrally to the skull using a denture base resin type II (Shanghai Medical Instruments Co., Ltd.). After surgery, the animals were housed individually on 12/12 h day/night schedule.

#### Signal acquisition

Following one week of recovery, the mice were connected to a headstage (HST/16D Gen2, Plexon Inc., Dallas, TX, USA) with the cable suspended by a helium balloon attached to a digital headstage processor (DHP/32), and allowed to freely explore a 40 × 40 cm arena for 30 min for habituation. Wideband signals were recorded using an OmniPlex Neural Recording Data Acquisition System (Plexon Inc., Dallas, TX, USA) with an 8 kHz global low-pass filter. The continuous spikes were sampled at 40 kHz, followed by a 300 Hz low-cut filter. The field potential (FP) was set to a 250 low-pass filter and down-sampled to 1 kHz. After recording, the hippocampus was post-fixed for Nissl staining to verify the proper placement of the electrodes in the target region.

#### Spectral analysis

The power spectrum density (PSD) and spectrogram of continuous FP were computed using NeuroExplorer (Nex Technologies, Colorado Springs, CO, USA) with 1024 frequency values and 25% window overlap. Before this process, the FP signal values were multiplied by the coefficients of the Hann window and discrete fast Fourier transformations of the results were calculated using formulas defined previously [22]. Theta/gamma waves were filtered by bandpass filtering of the FP data using NeuroExplorer software with Digital Filtering of Continuous Variables function. And amplitude-phase coupling analysis were performed using python script plugged in NeuroExplorer software. The modulation index (MIn) was calculated using the formula:$\,MIn=|{n}^{-1}\mathop{\sum }\limits_{t=1}^{n}{A}_{t}\,{e}^{i{\theta }_{t}}|$, where: *n* represents the total number of sample points, *t* indicates the time point, *A* denotes the amplitude/energy of the high-frequency band signal at time, *θ*_*t*_ represents the phase of the low-frequency band signal at time, *i* is the imaginary unit [23].

As PPT1-KI mice suffer from severe epilepsy at 6 ~ 7 months of age, this behavioral phenotype has been studied using the Racine classification method, please see our previously published article [24]. In this study, we monitor the field potential trace and create interval variables manually using NeuroExplorer when the field potential exhibits abnormal fluctuations. The criteria for identifying epileptiform activity refer to Dr. Nicolas G. Bazan’s studies: the epileptiform activity arising from the baseline with energy of the signal above 4 standard deviation, > 100 Hz, ≤ 4 sec of duration with bursting firing recorded in more than 4 contiguous channels (cover 400 ~ 500 μm of hippocampal area) [25]. Sharp-wave ripples (> 200 Hz) during the freely moving condition were also considered epileptiform activities. FP signals were acquired continuously for 1 hr per animal, with data stored as sequential 5-minute segments to optimize epileptiform discharge detection.

#### Spike sorting

Spikes were sorted using Offline Sorter (Plexon Inc., Dallas, TX, USA) to classify the electrical activity of individual neurons, based on the first to third principal components [26]. Spikes were identified when a minimum waveform reached an amplitude threshold of 3 standard deviations higher than the noise amplitude. Spike units were excluded when the absolute refractory period of single-unit autocorrelation was < 1 ms. Cross-channel artefacts identified by their time coincidence across channels were also invalidated. The recorded units were classified into fast-spiking INs and regular-spiking pyramidal cells based on mean discharge rates and spike waveform parameters (peak-valley ratio and half-valley width) using a Gaussian mixture model [27–29].

### Fiber photometry recordings

After anaesthesia, mice were stereotactically injected with the calcium-sensitive indicator rAAV-hSyn-GCaMP6s-WPRE-hGH-pA (Wuhan Pivotal Brain Science and Technology Co.) into the hippocampal CA1 region (coordinates relative to bregma: AP: -1.82 mm, ML: ±1.25 mm, DV: -1.5 mm) using a microinjection pump (model D-303376, Harvard Apparatus, USA) and a microsyringe, with an injection volume of 300 nL/hemisphere at a rate of 40 nL/min. Three weeks after injection, a fiber optic cannula with a 1.25 mm diameter ceramic ferrule and a 200 µm core, bundled with a protective sheath (R-FOC-BL200C-39NA, RWD, Shenzhen, China), was implanted 0.1-2 mm above the injection site. One week following implantation, the cannula was connected to a real-time fiber-optic recording system (R821, Reward Tri-Colour Multi-Channel Fibre-Optic Recording System, RWD, Shenzhen, China) to measure fluorescence changes. The light source illuminated the dye within the neurons, and the resulting fluorescence was captured by the fiber optic cannula.

To capture fluorescence signals, the fiber photometry system deployed two continuous sinusoidally modulated LEDs at 470 nm (excitation power 30 μW) and 410 nm (excitation power 15 μW) as light sources to excite GCaMP6s and an isosbestic autofluorescence signal, respectively. Upon excitation at 470 nm, GCaMP6s exhibited fluorescence intensity that was highly responsive to Ca2+ changes; however, when GCaMP6s was excited at 410 nm, the emission was largely Ca2 + -insensitive, which was used as a control.

Fluorescence changes were recorded in real-time during the experiment while the mice were moving freely. Once the mice entered the central zone of a 40×40 open field, the event were manually tagged. The raw fluorescence data were processed to correct for artifacts, such as movement or photobleaching. These corrections allowed for the calculation of changes in fluorescence intensity, serving as a proxy for calcium concentration changes, which were then analyzed to interpret the neuronal activity. We derived the values of GCaMP6s fluorescence change (ΔF/Fn) by calculating (F470 − F0)/F0, where F0 is the 2 min average baseline fluorescence of the F470 channel before event tag. The F410 channel is used as an isosbestic fluorescence channel; we derived the values of isosbestic fluorescence change (ΔF/Fn) by calculating (F410 − F0)/F0, where F0 is the 2 min average of the baseline fluorescence of the F410 channel before the event tag.

### Golgi-COX staining

Mice (6 to 7 months old) were briefly decapitated under anaesthesia with ethyl ether. Brain tissue was taken immediately and fixed for at least 24 h. The brain tissue was cut into tissue blocks with a thickness of 2-3 mm according to the tissue site to be observed, and rinsed several times with normal saline. Brain tissue was completely submerged in Golgi-COX staining solution and placed in a cool dark place for 14 days. The brain tissue was then placed in a 30% sucrose solution for 2 days at 4 °C in the dark. The brain tissue was removed and washed with distilled water for 1 min. Tissue slides were treated with concentrated ammonia water for 45 min, then washed with distilled water for 1 min, treated with fixing solution again for 45 min, and finally washed with distilled water for 1 min. The tissue slides were treated with 30% sucrose solution to dehydrate for 2 ~ 3 days at 4 °C, cut into 100 μm slices with an oscillating microtome, and pasted on a gelatin slide. The tissue slides were scanned using a 3DHistech Pannoramic 250 (Budapest, Hungary) and observed with Case Viewer (3DHistech). Neurons and glia appear black, and the background is greyish white or colorless.

Analyses of the density and number of dendritic spines were performed using Image J 6.0 (National Institutes of Health, Bethesda, MD) with a Sholl-analysis plugin [30, 31]. We observed the density of dendritic spines on the basilar dendrites in the 400× field of view by selecting the first branch of the dendrites from the cell body and calculated the dendritic spine number within the length range of 30 ~ 90 μm. The density of dendritic spines is determined by the number of dendrites per 10 μm.

### Immunostaining

Coronal hippocampal sections (3 μm thick) were prepared using a Leica RM2016 slicer (Germany). The sections underwent fixation, paraffin embedding, and were treated with xylene (15 min × 2) followed by a series of ethanol dehydration steps (5 min × 2 for each) using 100%, 85%, and 75% ethanol solutions, respectively. Slides were then rinsed with distilled water. For antigen retrieval, slides were immersed in EDTA buffer (pH 8.0) at a sub-boiling temperature for 8 min. After rinsing with PBS (pH 7.4, 5 min × 3), endogenous peroxidase activity was quenched by incubating the slides in 3% H_2_O_2_ for 25 min at room temperature (RT). Slides were then blocked using either 10% donkey serum (for primary antibodies derived from goat) or 3% BSA (for primary antibodies from other sources) for 30 min at RT. Primary antibodies were applied overnight at 4 °C in a humidified chamber. The primary antibodies used in this study were: anti-somatostatin (A9274, Abclonal), anti-parvalbumin (26521-1-AP, Proteintech), anti-cleaved-caspase-3 (GB11532-100, Servicebio), anti-GAD 65 (GB12562-100, Servicebio), anti-CaMKII-α (6G9) (#50049, Cell Signaling). After rinsing with PBS (pH 7.4, 5 min × 3), slides were incubated with species-appropriate secondary antibodies conjugated to HRP (e.g., HRP goat anti-mouse IgG (H + L) (AS003, Abclonal), HRP goat anti-rabbit IgG (H + L) (AS014, Abclonal) for 1 h at RT. Slides were then treated with TSA-FITC (Tyramide Signal Amplification with fluorescein isothiocyanate) for 10 min in the dark, followed by rinsing with TBST (5 min × 3). To remove any bound primary and secondary antibodies, slides were subjected to microwave treatment in EDTA buffer (pH 8.0) at a sub-boiling temperature for 8 min. Subsequently, slides were incubated with the second set of primary antibodies (diluted appropriately in PBS) overnight at 4 °C. After rinsing with PBS (pH 7.4, 5 min × 3), slides were incubated with species-appropriate secondary antibodies for 50 min in the dark at RT. To reduce spontaneous fluorescence, slides were treated with a fluorescence quenching reagent (G1221-5ML, Servicebio) for 5 min, followed by rinsing under running water for 10 min. Slides were then counterstained with DAPI for nuclear visualization and mounted using an anti-fade medium. The nuclei appeared blue, and positive cells were identified by green or red fluorescence depending on the fluorophore used.

Images were captured using a Leica microscope with a 40x objective lens and stored using CaseViewer software (3DHISTECH Ltd., Budapest, Hungary). The images were imported into Image J 1.52 d (National Institutes of Health, Bethesda, MD, USA) for further analysis. Each channel was split to isolate the signals from the respective fluorophores. Gaussian filters were applied to reduce noise, followed by background subtraction to minimize background fluorescence that could interfere with the analysis. To segment the cells of interest from the background, we applied thresholding to each channel. We utilized the Image Calculator tool in Image J to identify cells that are double positive for both markers. This was achieved by performing a logical ‘AND’ operation on the binary masks obtained from the thresholding step. After obtaining the double-positive mask, we conducted particle analysis in Image J to count the number of cells that were double labeled. The ‘Analyze Particles’ function was used with appropriate size and circularity settings to exclude debris and other artifacts.

### DAB staining

Coronal brain sections from 6 ~ 7 months old PPT1-KI mice and age-matched WT mice were utilized for DAB staining. The sections were initially washed with Tris-buffered saline (TBS), followed by antigen retrieval in a citric acid buffer at 70 °C for 20 min. After cooling, the sections were rinsed in TBS for 8 min and treated with 10% methanol and 3% H_2_O_2_ in TBS for 10 min to quench endogenous peroxidase activity.

The brain sections were then incubated with a primary antibody against NeuN (1:500 dilution, catalog number GB13138-1, Servicebio, Wuhan, China) overnight at 4 °C after blocking. Following this, the sections were rinsed in TBS for 8 min and incubated with a secondary horseradish peroxidase (HRP)-conjugated goat anti-mouse IgG (H + L) antibody (1:400 dilution in Supermix, Servicebio, Wuhan, China) for 1 h. The sections were subsequently incubated with 0.05% DAB to visualize the immunoreactivity.

Finally, the slides were mounted with micro cover slides for histological examination. Sections were analyzed using Case Viewer 2.0 software and Image J 1.52 d (National Institutes of Health, Bethesda, MD, USA) at a magnification of 200×, focusing on the hippocampal CA1 and CA3 regions.

### Statistics

All data were acquired and analyzed by researchers who were blinded to the genotype of the mice. All data were analyzed using SPSS Statistics 20 (IBM, Armonk, NY, USA). We assessed the homogeneity of variances using Levene’s test and checked for normality with the Shapiro-Wilk test. Data were statistically analyzed by two-side Student’s t-test for two-group comparisons and one-way ANOVA followed tukey’s multiple comparisons test or two-way ANOVA followed by Šídák’s multiple comparisons test for multi-group comparisons.

## Results

### Caspase activation in hippocampal PV+ interneurons reduces inhibition and drives pyramidal neuron hyperexcitability in PPT1 deficiency

**Fig. 1:**
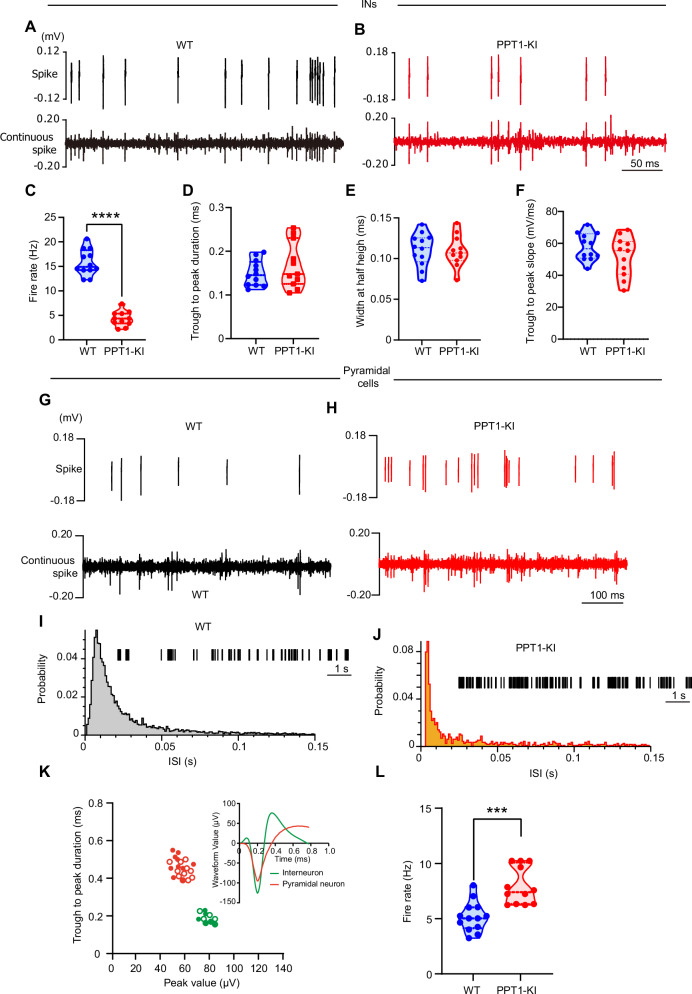
Reduced IN activity in PPT1-deficient mice disinhibits pyramidal cells at 3 ~ 4 months. **(A, B)** Sorted spike units and continuous spike signals from hippocampal INs in WT and PPT1-KI mice. **(C-F)** Comparison of firing rate **(C)**, trough to peak duration **(D)**, width at half height **(E)**, and trough to peak slope **(F)** of waveforms from INs in WT and PPT1-KI mice. WT: N = 12 neurons from 10 mice; PPT1-KI: N = 11 neurons from 11 mice; t-test: ****P < 0.0001. **(G, H)** Representative waveforms and continuous spike signals from hippocampal pyramidal cells in WT **(G)** and PPT1-KI **(H)** mice. **(I, J)** ISl histograms of pyramidal neurons recorded from WT **(I)** and PPT1-KI **(J)** mice. The spike unit sequence was displayed in the upper right corner. **(K)** Distribution of trough-to peak duration. Solid circles: WT, hollow circles: PPT1-KI. Insets display representative averaged waveforms of example INs and pyramidal neurons. **(L)** Comparison of firing rate of pyramidal neurons in WT and PPT1- KI mice. WT: N = 13 neurons from 11 mice; PPT1-KI: N = 12 neurons from 12 mice; t-test: ***P < 0.001.

To study the effects of PPT1 deficiency on the spike firing of INs, we implanted electrodes in the stratum pyramidale of the hippocampal CA1 region to record interneuronal spikes in mice. Through offline spike sorting, the firing of INs was well identified due to their distinctive waveform and frequency characteristics. In PPT1-deficient mice, the firing rate of hippocampal CA1 INs was significantly reduced compared to that of WT mice (two-tailed t-test, t_(21)_ = 12.55, ****P < 0.0001), with no significant differences observed in through-peak duration (two-tailed t-test, t_(21)_ = 1.045, *P* = 0.3078), width at half height (two-tailed t-test, t_(21)_ = 0.2052, *P* = 0.8394), and trough-peak slope (two-tailed t-test, t_(21)_ = 1.369, *P* = 0.1855) (Fig. 1A–F).

On the other hand, the firing rate of pyramidal neurons increased (two-tailed t-test, t_(23)_ = 4.563, ***P < 0.001) (Fig. 1G, H, L), and the inter-spike interval (ISI) histograms also showed that, compared to the WT group, the discharge intervals in the PPT1 group were shorter (Fig. 1I, J). The distinct waveforms of pyramidal neurons and INs were displayed in Fig. 1K. Compared to pyramidal neurons, INs had shorter wavelengths and longer distance from peak to trough (Fig. 1K).

Given that PV^+^ and SST^+^ neurons constitute the majority of INs in the hippocampal region, we postulate that the diminished firing rate of CA1 INs in PPT1-KI mice may stem from the reduced function or cell numbers (apoptosis) of the selected INs.

**Fig. 2:**
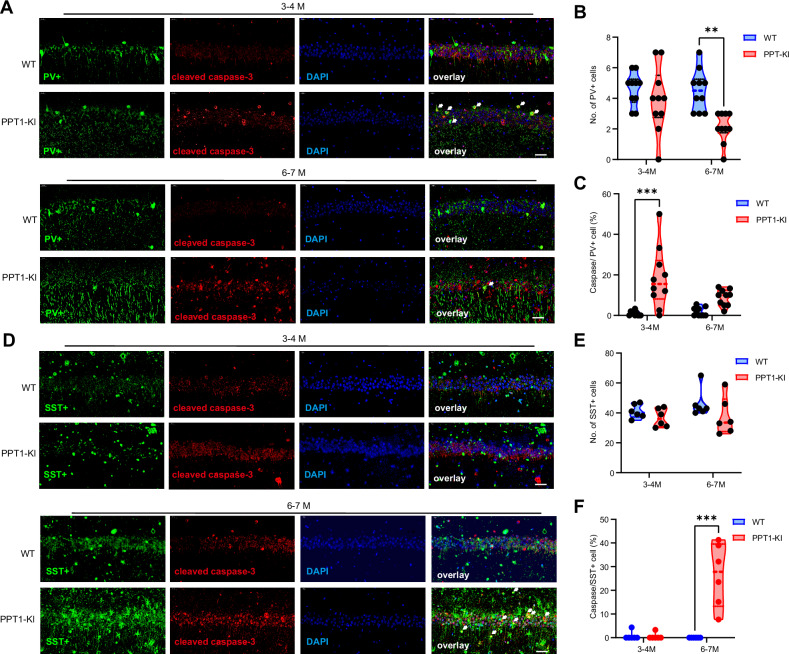
PPT1 deficiency induced the early and late activation of caspase 3 for PV^+^ and SST^+^ INs, respectively. **(A)** Immunostaining of PV positive (green) and cleaved caspase-3 positive (red) INs in CA1 regions of WT and PPT1-KI mice at 3 ~ 4 months old and 6 ~ 7 months old ages. White arrows denote cells that are double-positive for PV and cleaved caspase-3. **(B, C)** Comparison of number of PV positive cells **(B)** and double-positive for PV and cleaved caspase-3 **(C)** of WT and PPT1-KI at 3 ~ 4 months old and 6 ~ 7 months old ages. N = 10 for each group; two-way ANOVA, **P < 0.01, ****P < 0.001. **(D)** Immunostaining of SST positive (green) and cleaved caspase-3 positive (red) INs in CA1 regions of WT and PPT1-KI mice at 3 ~ 4 months old and 6 ~ 7 months old ages. White arrows denote cells that are double-positive for SST and cleaved caspase-3. **(E, F)** Comparison of number of SST positive cells **(E)** and double-positive for PV and cleaved caspase-3 **(F)** of WT and PPT1-KI at 3 ~ 4 months old and 6 ~ 7 months old ages. N = 6 for each group; two-way ANOVA, ****P < 0.0001. Scale bar: 40 μm.

Immunofluorescent labeling showed a significant increase in PV^+^/cleaved-caspase 3+ co-labeled cells in the hippocampus of 3 ~ 4 months old PPT1-KI mice, compared to WT controls (two-way ANOVA, t(36) = 1.072, P = 0.49). Furthermore, examination of pyramidal neurons, labeled with CaMKIIα, revealed no co-localization with cleaved-caspase 3 in either WT or PPT1-KI mice at this same stage (Fig. S2A). No difference in total PV^+^ interneuron counts was observed between genotypes. However, a subsequent significant reduction of PV^+^ INs by 6 ~ 7 months of age was seen in PPT1-KI mice (6 ~ 7 M: two-way ANOVA, t_(36)_ = 3.677, **P < 0.0015) (Fig. 2A–C), suggesting that at 3 ~ 4 months, PV+ INs in the hippocampus of PPT1-KI mice are undergoing caspase 3 activation (3 ~ 4 M: two-way ANOVA, t_(36)_ = 4.986, ***P < 0.001), culminating in a decreased PV^+^ IN population by 6 ~ 7 months (6 ~ 7 M: two-way ANOVA, t_(36)_ = 2.010, *P* = 0.1013). On the other hand, the neither caspase 3 activation (two-way ANOVA, t_(20)_ = 0.04311, *P* = 0.9988) nor cell count change of SST^+^ INs in PPT1-KI mice was observed at 3 ~ 4 months (two-way ANOVA, t_(20)_ = 0.8583, *P* = 0.6411). However, an increase in the number of cells expressing both SST and cleaved-caspase 3 was observed at 6 ~ 7 months of PPT1-KI mice (two-way ANOVA, t_(20)_ = 6.747, ***P < 0.001) (Fig. 2D–F).

We also examined GAD65 expression in PPT1-KI mice at different ages. Immunofluorescence staining and Western blot analysis confirmed that, compared to WT, GAD65 expression in the hippocampal CA1 region was significantly reduced in 6- to 7-month-old PPT1-KI mice (two-tailed t-test, t_(4)_ = 2.853, *P = 0.0462), but not in 3- to 4-month-old mice (two-tailed t-test, t_(4)_ = 0.4847, *P* = 0.6533) (Fig. S3).

**Fig. 3:**
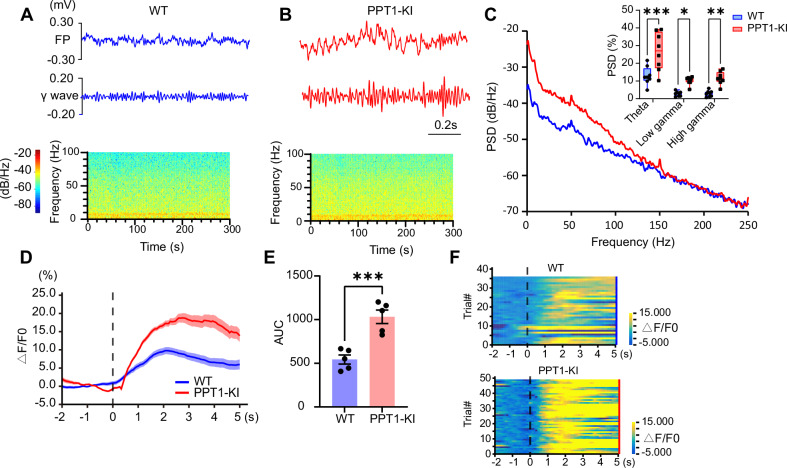
Enhanced neuronal calcium activity and theta/gamma oscillatory power in the CA1 region of the hippocampus of 3 ~ 4 months old PPT1-KI mice. **(A, B)** FP signals and spectrograms recorded in the CA1 region of WT **(A)** and PPT1-KI **(B)** (3 ~ 4 months old). **(C)** PSD of FP recorded from WT (blue) and PPT1-KI (red) mice. The upper right panel illustrates the analysis of PSD across theta (3 ~ 8 Hz), low gamma (30 ~ 50 Hz), and high gamma (50 ~ 90 Hz) frequency bands. N = 8 mice for each group, two-way ANOVA, **P < 0.01, ***P < 0.001. **(D)** The ΔF/F0 curve that indicates the change in fluorescence signals (ΔF/F0) from 2 seconds before to 5 seconds after the time point of entering the central area. Blue curve represents mean values for WT animals ± SEM (shaded area). Red curve represents mean values for PPT1-KI animals ± SEM (shaded area). N = 5 mice. **(E)** Mean area under the curve (AUC) from curves presented in **(D)** for animals tested under the freely moving condition. N = 5 for each group, t-test, ***P < 0.001. Data represented as mean ± SEM. **(F)** Heat maps illustrating the change in fluorescence signals (ΔF/F0) from 2 seconds before to 5 seconds after the time point of entering the central area.

Subsequently, we aim to explore the consequences of impaired interneuronal activity resulting from PPT1 deficiency on the neural network’s activity. In vivo FP analysis revealed that the absence of PPT1 enhances the PSD of theta (two-way ANOVA, t_(42)_ = 5.211, ***P < 0.001) and low/high gamma oscillations in the hippocampal FPs of 3 ~ 4 months old mice (two-way ANOVA, low gamma: t_(42)_ = 2.839, *P = 0.0207; high gamma: t_(42)_ = 3.352, **P = 0.0051) (Fig. 3A–C). Additionally, fiber photometry recordings demonstrated that, compared to WT mice, the neuronal calcium response in the hippocampal CA1 region of 3 ~ 4 months old PPT1-KI mice is increased (two-tailed t-test, t_(8)_ = 5.254, ***P < 0.001) (Fig. 3D–F). Furthermore, we observed that PPT1 deficiency attenuates coupling between the theta phase and gamma amplitude in the hippocampus compared with WT controls. Diazepam treatment partially reinstates the coupling relationship between these two oscillation components (one-way ANOVA, WT vs. PPT1-KI, F_(2, 15)_ = 9.372, **P = 0.0025; PPT1-KI vs. PPT1-KI + D.Z., *P = 0.0145) (Fig. S4 A–E).

These findings indicate that PPT1 deficiency triggers caspase 3 activation in mouse hippocampal INs, leading to excessive activation of neurons and disrupting theta-gamma phase-amplitude coupling (PAC).

### PPT1 deficiency leads to hippocampal seizure-like discharges and associated ripple oscillation

**Fig. 4:**
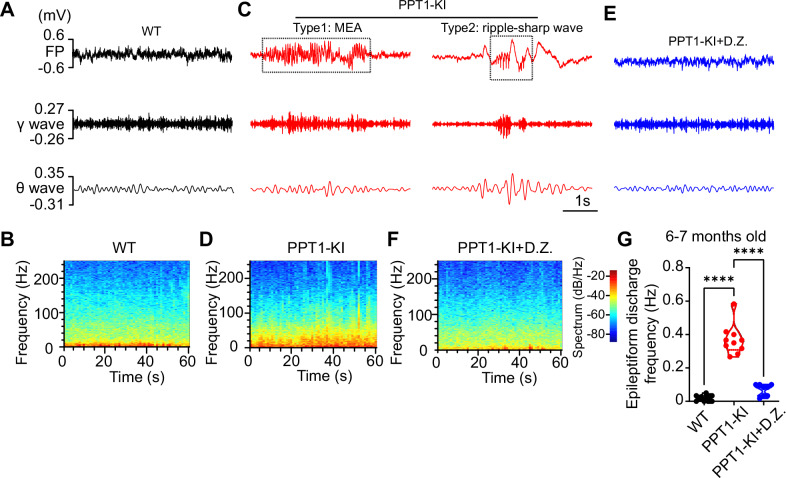
Epileptiform activities in the CA1 region of 6 ~ 7 months old PPT1-KI mice. **(A-F)** LFP signals and spectrograms recorded at CA1 region from WT **(A** and **B)**, PPT1-KI **(C** and **D)**, and PPT1-KI treated with D.Z. **(E** and **F)** mice (6 ~ 7 months). The lower traces showing filtered theta (4 ~ 12 Hz) and gamma oscillation (30 ~ 80 Hz) from LFP signals. The dashed line box in B indicated epileptiform discharges detected from CA1 region from PPT1-KI mice. MEA: microepileptiform activity. **(G)** Analysis of epileptiform frequency. WT: n = 13 mice; PPT1-KI: n = 10 mice; PPT1-KI + D.Z.: n = 8 mice, One-way ANOVA, ***P < 0.001.

A previous study suggested that seizures could be detected after 7 months in PPT1-/- mice using electroencephalogram recordings [32]. Consistently, using in vivo FP recording with spectral analysis, we also observed two types of epileptiform discharges in the hippocampal CA1 region at 6 ~ 7 months old but not in 3 ~ 4 months old PPT1-KI mice (Fig. 4A–D), intraperitoneal injection of diazepam exhibited a suppressive effect on epileptic activity compared with untreated PPT1-KI mice (one-way ANOVA, F_(2,28)_ = 91.80, WT vs. PPT1-KI, ***P < 0.001; PPT1-KI vs. PPT1-KI + D.Z., ***P < 0.001) (Fig. 4E–G).

Previous studies also indicated that following the onset of temporal lobe epilepsy, a diminished frequency of physiological ripples has been implicated in the animal’s failure to effectively consolidate spatial memory [33]. A link of ripples to epilepsy might be suggested since they also appear in recordings from epileptic foci [34, 35]. These evidences prompt us to explore the impact of the dysregulation of palmitoylation modification caused by PPT1 deficiency on the occurrence of epilepsy and its effects on physiological ripple oscillations in the hippocampus.

**Fig. 5:**
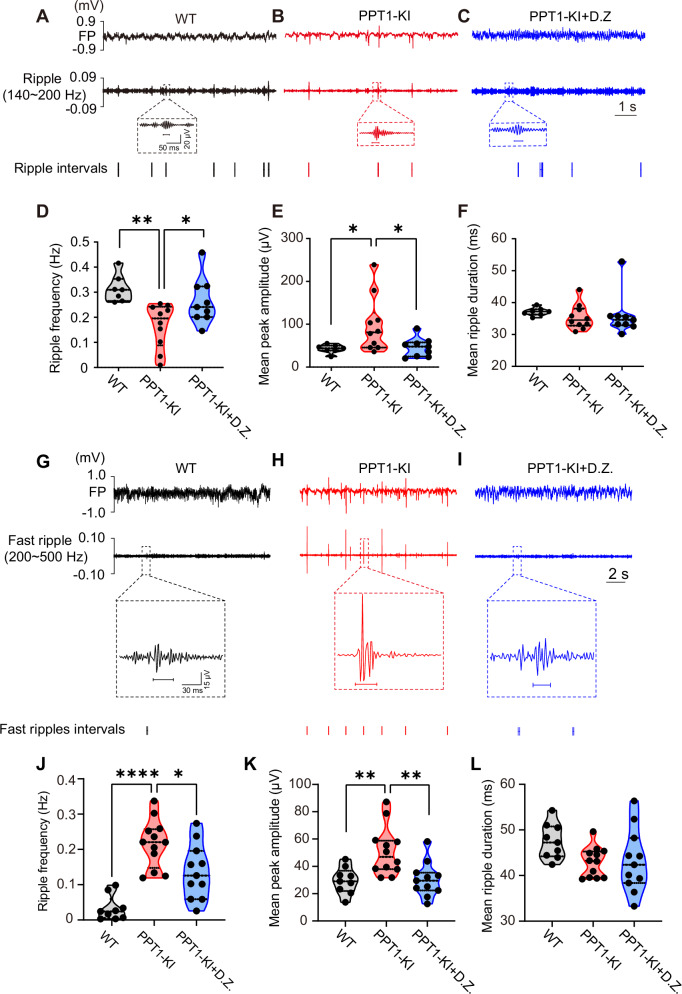
Hippocampal ripple oscillations show decreased frequency but increased amplitude while fast ripple oscillations exhibit increased frequency and amplitude in 6 ~ 7 months old PPT1-KI mice. **(A-C)** LFP signals recorded at CA1 region from WT **(A)**, PPT1-KI **(B)**, and PPT1-KI treated with D.Z. mice **(C)** (6 ~ 7 months) under freely moving conditions. The lower traces showing filtered ripple oscillation (140 ~ 200 Hz) from LFP signals. The dashed line boxes display enlarged ripple oscillations detected in the CA1 region of WT, PPT1-KI, and PPT1-KI mice treated with D.Z. **(D-F)** Analysis of ripple frequency **(D)**, mean peak amplitude **(E)**, and mean ripple duration **(F)**. WT: n = 7 mice; PPT1-KI: n = 10 mice; PPT1-KI + D.Z.: n = 9 mice. One-way ANOVA, *P < 0.05, **P < 0.01. **(G-I)** LFP signals recorded at CA1 region from WT **(G)**, PPT1-KI **(H)**, and PPT1-KI treated with D.Z mice **(I)** (6 ~ 7 months) under freely moving conditions. The lower traces showing filtered fast ripple oscillation (200 ~ 500 Hz) from LFP signals. The dashed line boxes display enlarged fast ripple oscillations detected in the CA1 region of WT, PPT1-KI, and PPT1-KI mice treated with D.Z. **(J-L)** Analysis of fast ripple frequency **(J)**, mean peak amplitude **(K)**, and mean ripple duration **(L)**. WT: n = 9 mice; PPT1-KI: n = 12 mice; PPT1-KI + D.Z.: n = 11 mice. One-way ANOVA, *P < 0.05, **P < 0.01,****P < 0.0001.

As shown in Fig. 5A–E, alongside the epileptiform discharges, the PPT1-KI mice exhibited an augmentation in the amplitude and a reduction in the frequency of physiological ripple oscillations (140 ~ 200 Hz). However, there was no significant change observed in the mean ripple duration (Fig. 5F). The administration of diazepam effectively elevates the frequency and reduces the amplitude of ripple oscillations (frequency: one-way ANOVA, F_(2, 23)_ = 7.104, WT vs. PPT1-KI, **P = 0.04; PPT1-KI vs. PPT1-KI + D.Z., *P = 0.0456; amplitude: one-way ANOVA, F_(2, 23)_ = 4.694, WT vs. PPT1-KI, *P = 0.0423; PPT1-KI vs. PPT1-KI + D.Z., *P = 0.0393; duration: one-way ANOVA, F_(2, 23)_ = 0.2580, WT vs. PPT1-KI, *P* = 0.7597; PPT1-KI vs. PPT1-KI + D.Z., *P* = 0.9738), suggesting a therapeutic potential for diazepam in rectifying the neural oscillation disruptions caused by PPT1 deficiency.

As fast ripples (200 ~ 500 Hz) are intimately associated with the progression of epilepsy [36, 37], we next examined whether PPT1 deficiency alters these oscillations. Our in vivo electrophysiological experiments demonstrated that in PPT1-KI mice at 6 ~ 7 months of age, the frequency and amplitude of fast ripple oscillations in the hippocampal CA1 region are significantly increased compared to those in WT mice. Treatment with diazepam effectively suppresses the enhancement of both frequency and amplitude of fast ripples (frequency: One-way ANOVA, F_(2, 29)_ = 20.67, WT vs. PPT1-KI, ****P < 0.0001; PPT1-KI vs. PPT1-KI + D.Z., *P = 0.027; amplitude: One-way ANOVA, F_(2, 29)_ = 8.173, WT vs. PPT1-KI, **P = 0.0052; PPT1-KI vs. PPT1-KI + D.Z., **P = 0.0045; duration: One-way ANOVA, F_(2, 29)_ = 2.601, WT vs. PPT1-KI, *P* = 0.1092; PPT1-KI vs. PPT1-KI + D.Z., *P* = 0.9899) (Fig. 5G–L).

After electrophysiological recordings, the expression of the hippocampal GABA_A_R α1 subunit was analyzed using Western blot. We found that GABA_A_R α1 expression was significantly reduced in 6- to 7- months-old PPT1-KI mice compared to WT littermates. Diazepam treatment did not rescue this reduction and was associated with a slight decrease in GABA_A_R α1 expression (one-way ANOVA, F(2,6) = 10.44, WT vs. PPT1-KI, *P < 0.05; PPT1-KI vs. PPT1-KI + D.Z., ns: no significant difference; WT vs. PPT1-KI + D.Z.,*P < 0.05) (Fig. S5), consistent with the known ligand-induced downregulation of GABA_A_R α1 [38]. These results suggest that diazepam’s functional efficacy in suppressing seizures and restoring oscillations is primarily mediated through allosteric potentiation of existing receptors, rather than through altered expression of GABA_A_R subunit.

### Decreased neuronal excitability and morphological degeneration in late-stage PPT1 deficiency mouse model

**Fig. 6:**
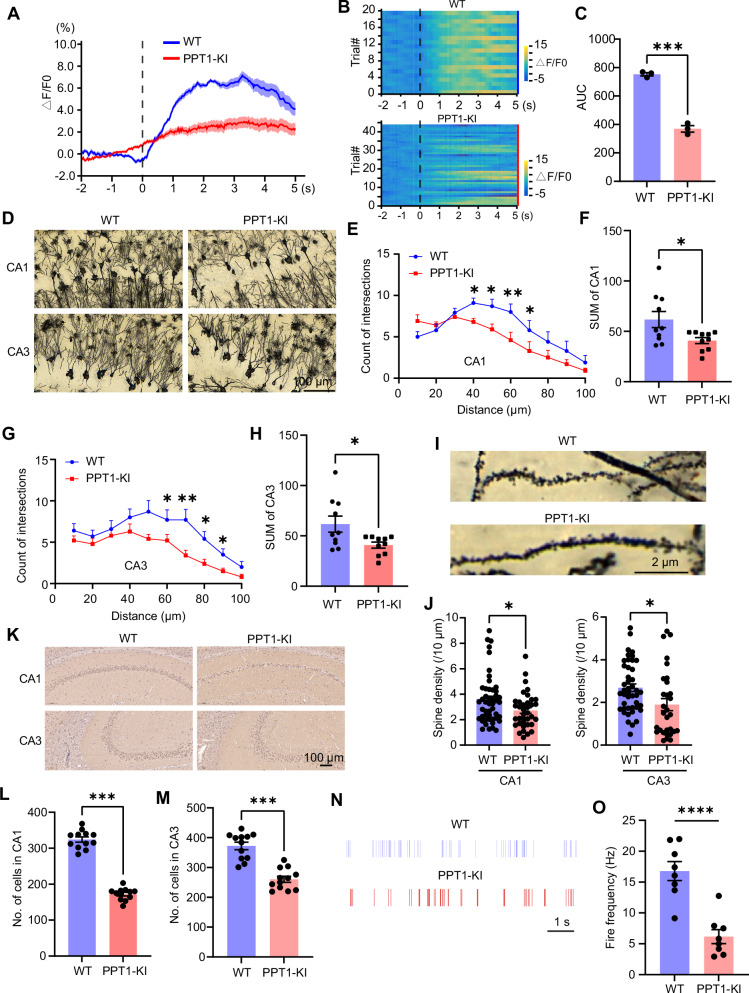
PPT1 deficiency leads to reduced hippocampal neuronal excitability and neuron loss in 6 ~ 7 months old mice. **(A-C)** Averaged ΔF/F0 curves **(A)**, heatmap **(B)**, and AUC **(C)** comparison of hippocampal activity in WT and PPT1 mice. N = 3 mice for each group, t-test, ***P < 0.001. AUC: area under the curve. **(D)** Sample images of Golgi-stained hippocampal neurons from WT and PPT1-KI mice. **(E,F)** Sholl analysis **(E)** and sum of intersections **(F)** of neurons in CA1 area**. E**: two-way ANOVA, *P < 0.05, **P < 0.01; **F:** t-test, *P < 0.05. N = 10 cells for each group. **(G,H)** Sholl analysis **(G)** and sum of intersections **(H)** of neurons in CA3 area**. G**: two-way ANOVA, *P < 0.05, **P < 0.01; **H:** t-test, *P < 0.05. N = 10 cells for each group. **(I,J)** Representative Golgi-stained image of dendritic spines **(I)** and spine density analysis **(J)** of basilar dendrites in hippocampal CA1 and CA3 pyramidal neurons. WT: n = 48 dendrites from 3 mice; PPT1-KI: n = 34 dendrites from 3 mice, t-test, *P < 0.05. **(K-M)** Sample images **(K)** and analysis of DAB staining in hippocampal CA1 **(L)** and CA3 **(M)** at 200×magnification. N = 12 ROIs for each group, t-test, ***P < 0.001. **(N-O)** Raster plot of spike units sorted from hippocampal CA1 pyramidal neuron and **(N)** count of spike number per 5 min **(O)**. N = 9 spike units for each group, t-test, ****P < 0.0001.

In vivo fiber photometry recordings revealed that PPT1-KI mice exhibited reduced calcium signaling activity in hippocampal neurons at 6 ~ 7 months of age compared to age-matched WT littermates (two-tailed t-test, t_(4)_ = 15.05, ***P < 0.001) (Fig. 6A–C).

We next employed Golgi staining to observe morphological changes in hippocampal neurons of PPT1-KI mice (Fig. 6D). Sholl analysis results indicated a decrease in dendritic branching (Fig. 6E–H) (E and G: two-way ANOVA, *P < 0.05, **P < 0.01; F and H: two-tailed t-test, t_(18)_ = 2.108, *P < 0.05) and spine density (two-tailed t-test; CA1: t_(83)_ = 2.24, *P < 0.05, CA3: t_(72)_ = 2.47, *P < 0.05) (Fig. 6I, J) in the hippocampal CA1 and CA3 regions of PPT1-KI mice. Additionally, DAB staining with antibody targeting NeuN showed a reduction in neuronal count (CA1, two-tailed t-test, t_(22)_ = 17.44, ***P < 0.001; CA3, t_(22)_ = 6.891, ***P < 0.001)(Fig. 6K–M), in vivo electrophysiological recording also indicated a reduction in the number of spike units in the CA1 area of the hippocampus in PPT1-KI mice at 6 ~ 7 months of age (two-tailed t-test, t_(14)_ = 5.54, ****P < 0.0001) (Fig. 6N,O).

These findings indicate that late-stage pathology of *CLN1* disease in PPT1-KI mice is characterized by extensive neuronal loss in the hippocampus, which extends beyond INs to include the majority of excitatory pyramidal neurons.

## Discussion

**Fig. 7:**
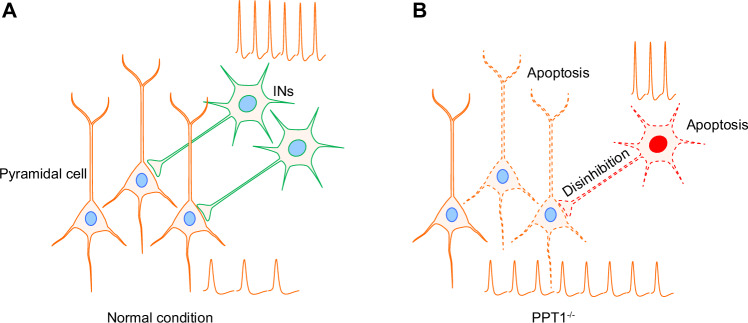
Schematic diagram demonstrates that PPT1 deficiency induces IN apoptosis, leading to disinhibition of pyramidal cells. **A** Feedback inhibition of pyramidal neuron activity by INs in the hippocampal region. **B** PPT1 deficiency leads to IN apoptosis, resulting in disinhibition of pyramidal neurons and subsequent network hyperexcitability.

Our studies indicate that in the PPT1-deficient model simulating *CLN1* disease, the early stages of disease onset are characterized by a loss function of PV^+^ INs, which leads to a failure in their inhibitory control over pyramidal neurons, excessive excitation of pyramidal neurons and a disruption of CFC within neural networks, ultimately the occurrence of epilepsy, as shown in Fig. 7. Persistent epileptic symptoms may indiscriminately cause neuronal loss and diminish neural activity in the later stages of the disease, culminating in atrophy of the hippocampus and potentially the entire brain. The dysfunction observed at the different age stages of the PPT1-KI mice is summarized in Table 1.

**Table 1:** Phenotype Changes of PPT1-KI Mice with Age.

Age Stage (Months)	Disfunction Symptoms
3-4 M	Interneuron activity reduction, neuronal calcium activity enhancement, theta/gamma oscillatory power enhancement, APC impairment, caspase-3 positivity in PV+ INs increase
6-7 M	Reduction in the number of PV+ INs, caspase-3 positivity in SST+ INs increase, epileptiform discharges, ripple frequency reduction, fast ripple frequency increase, neuronal calcium activity decrease, neuronal loss, spine number reduction, spike firing rate reduction

Our research proposes potential therapeutic strategies for the intervention of *CLN1* disease, which is to improve the function of inhibitory INs via inhibition of caspase activation in early stage and prevent late-onset seizure and neuronal apoptosis.

### PPT1 deficiency and caspase 3 activation/apoptosis of PV^+^/SST^+^ INs

Distinct subtypes of inhibitory INs are known to shape diverse rhythmic activities in the cortex. PV^+^ INs fire at high frequency with high reliability, enabling them to execute the encoding, consolidation and retrieval of memories as well as control network oscillations [39]. PV^+^ INs are instrumental in the synchronization with pyramidal neurons facilitating the emergence of gamma oscillations via rapid inhibitory actions [40–42]. Conversely, SST^+^ INs exhibit a more intimate association with theta-beta oscillations [17, 43], revealing differential and cooperative roles of SST^+^ and PV^+^ inhibitory neurons in orchestrating specific cortical oscillations [44].

Correlated with its role in modulating cortical oscillations, somatostatin IN subtypes form cell-type-specific circuits [45] and contribute memory function [46].

Recent study revealed that SST^+^ INs paradoxically facilitated seizure generalization, but PV^+^ INs retained an inhibitory role, suggesting that the SST^+^ INs play an excitatory role in the dentate gyrus which drive a widespread seizure network in cortical dysplasia [47].

In our study, we discovered for the first time that the deficiency of PPT1 leads to the caspase activation of PV^+^ INs, reduced firing of INs and disrupted hippocampal theta-gamma CFC in the 3 ~ 4 months old PPT1-KI mice correlated to the early stage of *CLN1* disease, indicating the impaired function of inhibitory neurons. It is thus not surprising that increased excitability of pyramidal neurons can be observed in this early stage of PPT1-KI mice. These events emphasize that selective PV^+^ INs dysfunction is likely a crucial player in the early pathology underlying *CLN1* disease. We propose that the selective vulnerability of PV^+^ INs may stem from their unique high metabolic demand as fast-spiking neurons [48–50]. PPT1 deficiency likely induces network hyperexcitability. This sustained high-frequency firing could push their already substantial energy requirements to the point of metabolic exhaustion, thereby triggering their preferential caspase-3 activation and initiating the early pathology of *CLN1* disease.

Neither caspase 3 activation nor cell number change can be observed in hippocampal SST^+^ INs in the early stage of PPT1-KI mice, indication of the relatively preserved function of SST^+^ INs in PPT1-KI mice. However, based on impaired function of PV^+^ INs, it is unlikely that SST^+^ INs is responsible for the enhanced power of theta and/or gamma in the early stage of PPT1-KI mice. Interestingly, we recently reported the elevated inhibitory inputs to CA1 pyramidal neurons in PPT1-KI animals, which may contribute to the enhancement of network oscillations such as theta and gamma oscillations [51]. The elevated inhibitory inputs might compensate for the loss of PV^+^ INs function.

Our data showed caspase activation with preserved cell numbers in PV^+^ INs in PPT1-KI mice at 3 ~ 4 months, which is consistent with studies that show caspase activation resulted in local pruning of dendritic spines and dendritic retraction via a caspase-3-dependent mechanism without neuronal death [52, 53]. Given the caspase activation in PV^+^ INs, it is difficult to interpret the enhanced theta and gamma oscillations observed in PPT1-KI mice at 3 ~ 4 months. However, SST^+^ INs also play a critical role in maintaining network function [44] and no caspase activation was observed in this type of INs in PPT1-KI mice at 3 ~ 4 months. Therefore, whether SST^+^ INs play a compensatory role in maintaining abnormal high gamma oscillations remains to be determined.

By 6 ~ 7 months, due to massive cell apoptosis, including both INs and pyramidal neurons, it is undeniable that theta and gamma oscillations will weaken. The remaining pyramidal neurons, lacking the inhibitory effect of INs, exhibit sustained epilepsy and abnormally increased fast ripple oscillations.

### Ripple oscillation, seizure, and PPT1

Ripple oscillation, also named high frequency oscillations (> 80 Hz) is an important type of neural network oscillation widely present in the hippocampal region. The generation mechanism of ripple oscillations in the hippocampus refers to a complex interaction of excitatory and fast-spiking inhibitory INs activities [54]. Ripple oscillations are categorized into physiological ripples (140 ~ 200 Hz) [55, 56] and pathological ripples (150 ~ 500 Hz) [36]. The physiological ripples are also associated with learning process and memory encoding [57–60], while the fast pathological ripples are closely linked to the development of epilepsy. They appear at the onset of epileptic events [36, 37, 61, 62], and therefore have diagnostic value in that they can be used to predict and localize epileptic seizures [63].

In our studies, when performing in vivo FP recordings in the PPT1-KI mouse, we observed that the frequency of ripple oscillations was significantly reduced compared to WT mice, while their amplitude was increased. This finding was in agreement with previous study that show seizures consistently occurred during intervals of increased ripple amplitude and decreased ripple frequency [64]. Combining Golgi staining results, we found an overall reduction in hippocampal neurons in the late stages of PPT1-KI mouse model. This suggests that the decrease in physiological ripple frequency may be associated with a decrease in the total number of cells in PPT1-KI hippocampus.

For fast ripples, our study results indicate that, compared to WT mice, PPT1-KI mice show an increase in both the frequency and amplitude of fast ripple oscillations in the hippocampal CA1 region, indicated that in PPT1-KI mice, disinhibition from INs to pyramidal neurons in the hippocampus may lead to excessive synchronization of pyramidal neuron firing, resulting in enhanced pathological fast ripples and the onset of epilepsy.

Indeed, PV^+^ interneurons typically contribute to the synchrony of excitatory neurons under physiological conditions. However, hyperexcitability and hypersynchrony of neuronal networks due to interneuron dysfunction are thought to be linked to the generation of epileptic activity, such as interictal-like discharges, in both humans and animal models [65, 66]. Consistent with this, studies show that despite a decrease in PV^+^ cell numbers, the remaining perisomatic synapses exhibit larger active zones [67] or dendrite sprouting [68], indicating stronger inhibition per synapse. This may enable more coordinated control of pyramidal cells and facilitate pathological hypersynchronization. Therefore, we speculate that the enhancement of fast ripple oscillations may be associated with interneuron dysfunction.

For the first time, we have identified the disruption of ripple oscillations in a mouse model deficient in PPT1 enzyme, suggesting that the depalmitoylation modification of proteins plays a crucial role in maintaining neural network activity. These findings provide new insights into the pathophysiological mechanisms underlying the altered ripple dynamics in the context of PPT1 deficiency and its implications for epilepsy development.

### Diazepam’s therapeutic potential in PPT1-deficient mice

Diazepam is a benzodiazepine that acts as an agonist at GABA_A_ receptors [69], exerting anxiolytic [70], sedative [71, 72], muscle-relaxant [73], and anticonvulsant, as well as anti-epileptic effects [74, 75]. Most of these effects are thought to result from a facilitation of the action of GABA [76–79], an inhibitory neurotransmitter in the central nervous system. In this study, we introduced, for the first time, the use of intraperitoneal injection to treat epilepsy in PPT1-deficient mice. Our findings indicate that acute diazepam administration was effective in suppressing the emergence of seizure-like discharges and partially restoring PAC between theta and gamma oscillations, as well as the frequency of ripple oscillations. Although there are currently no effective treatments for *CLN1* disease [80], our animal experimental results indicate that diazepam can suppress epileptic symptoms in the late stage of *CLN1*.

However, the reduction of epileptiform activity observed after acute diazepam administration in the progressive stage is not unexpected, given its non-specific suppression of neuronal activity. More targeted experiments, such as a conditional knock-in selectively targeting GABAergic neurons, are needed to further verify the mechanism. Our results at least demonstrate that non-specific activation of GABA receptors can suppress epileptiform activity in the late stage of *CLN1* disease.

These results suggest that the deficiency of GABAergic INs, particularly the loss of PV^+^ and SST^+^ neurons, and the subsequent failure of inhibitory modulation of pyramidal neurons, might be involved in the principal pathological mechanism underlying epilepsy in PPT1-deficient animal models. Future approaches aiming to enhance GABAergic inhibition may offer novel therapeutic targets and strategies to alleviate epileptic symptoms caused by *CLN1* mutation.

## Supplementary information

Suppl. information

## Data Availability

Data are made available from the corresponding author upon request.
