# Supplementary material for: Dysfunction of GABAergic interneurons underlies altered neural network oscillations associated with epileptiform activity in PPT1-deficient mice
Source: Transl Psychiatry. 2026 Feb 2;16:106. doi: 10.1038/s41398-026-03843-8 (PMC12923735; doi:10.1038/s41398-026-03843-8)
Supplement: Supplementary file 1 — Suppl. information [file 41398_2026_3843_MOESM1_ESM.docx]

Supplementary Materials for

**Dysfunction of GABAergic interneurons underlies altered neural network oscillations associated with epileptiform activity in PPT1-deficient mice**

***Supplemental figures and legends***

**
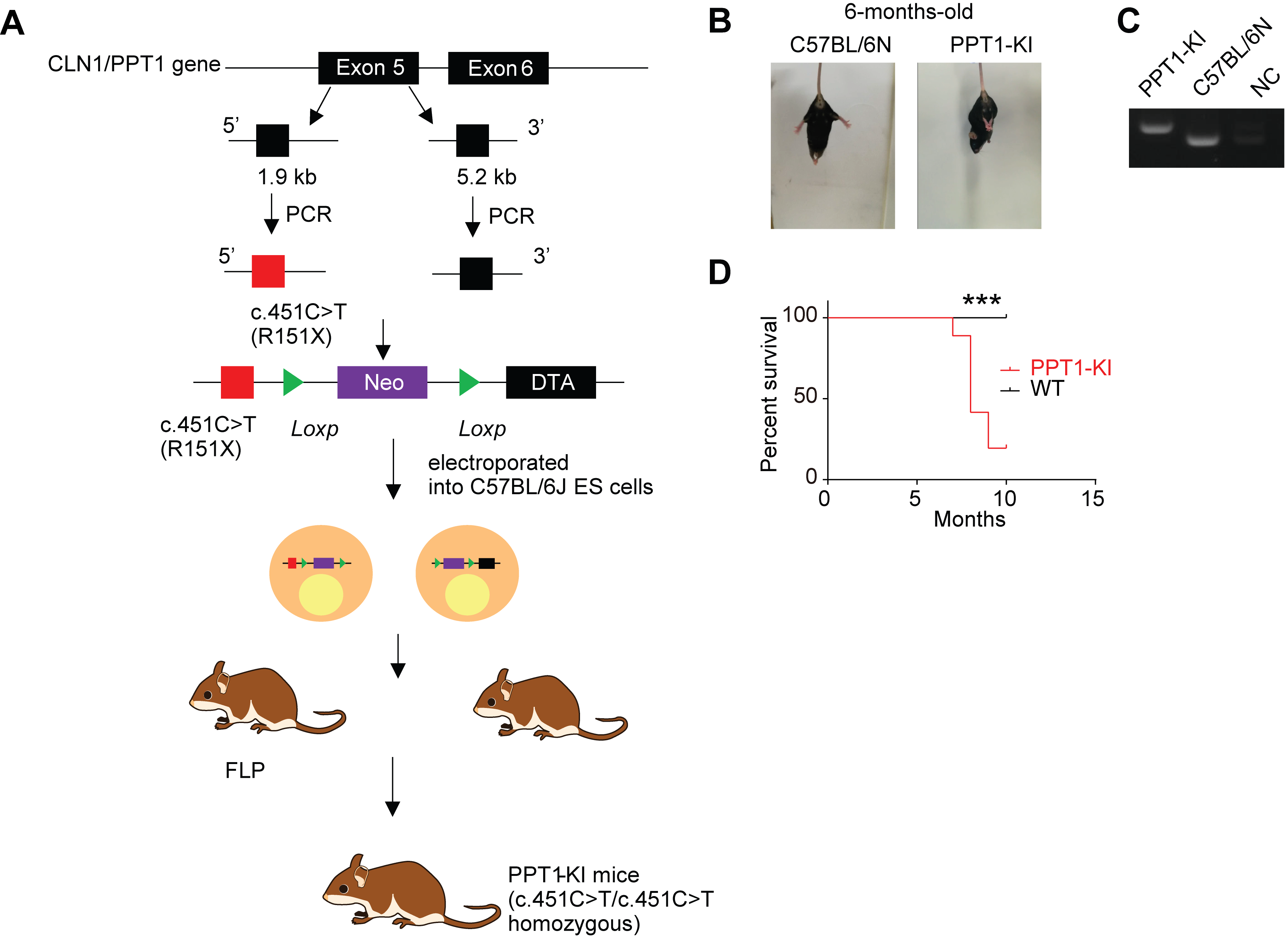
**

**Fig. S1 The strategy of point mutation of *CLN1* to generate PPT1-KI (c.451C>T) mice**

**(A)** The process of point mutation in the Cln1 c.451C>T nonsense mutation. ES cell, embryonic stem cell.

**(B)** Clasping behavior of 6-month-old WT and PPT1-KI mice.

**(C)** Representative blots of Cln1 gene identification. NC, negative control.

**(D)** Survival curves of PPT1-KI and WT mice. Chi-squared test = 46.21, df = 1, ****P* < 0.001.


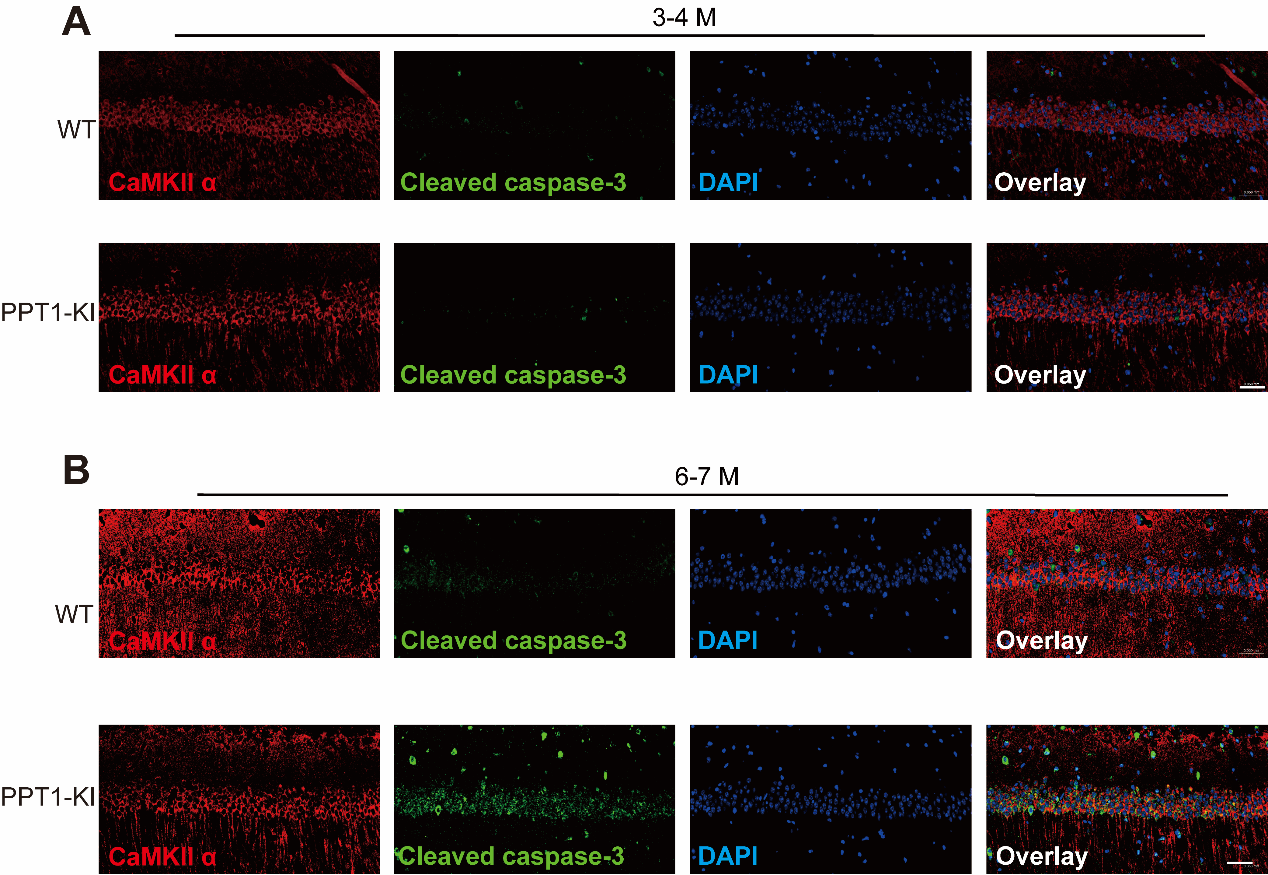


**Fig. S2 Absence of caspase-3 activation in pyramidal neurons in the CA1 region of PPT1-KI mice at the early stage**

**(A-B)** Immunostaining of CaMKII α positive (red) and cleaved caspase-3 positive (green) pyramidal neurons in CA1 regions of WT and PPT1-KI mice at 3~4 months old **(A)** and 6~7 months old **(B)** ages. Scale bar: 50 μm.

**
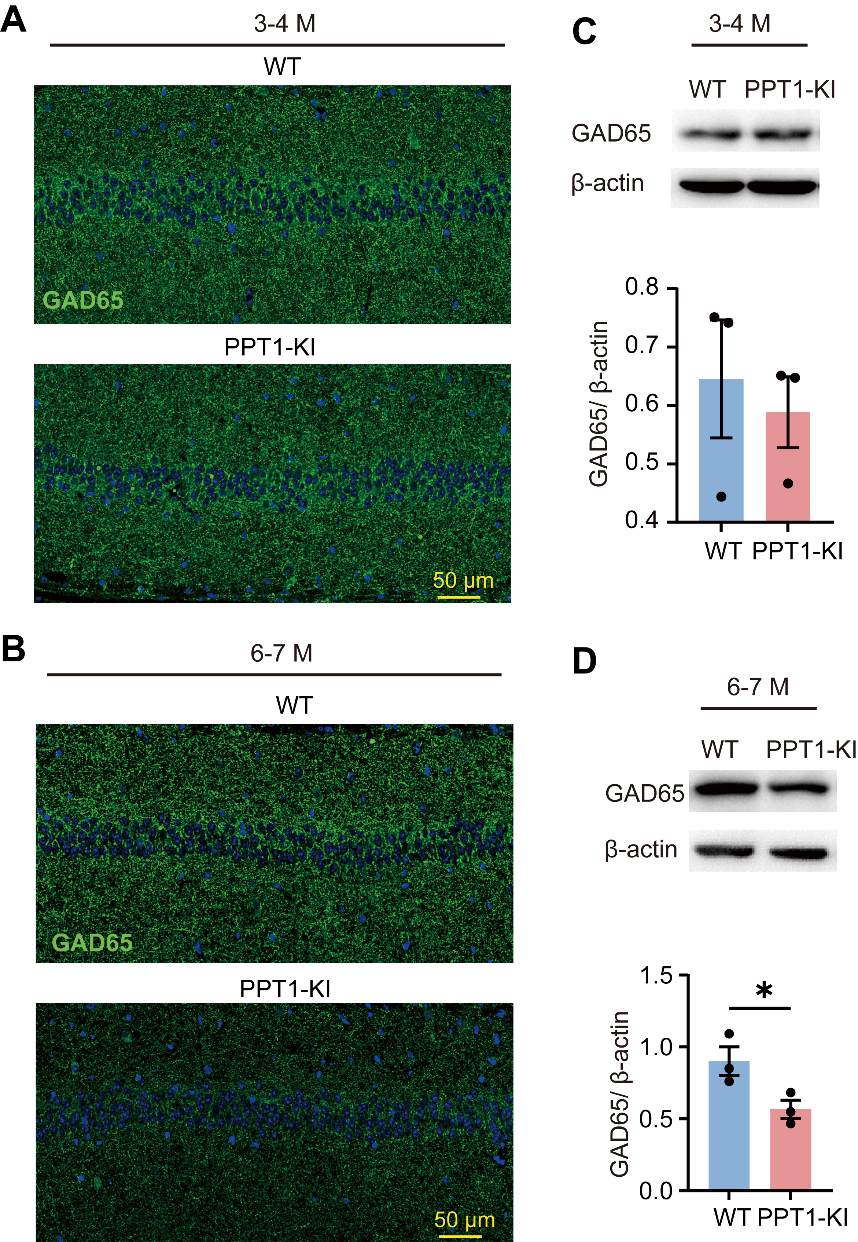
**

**Fig. S3 GAD65 expression in the hippocampal CA1 region of PPT1-KI mice at different ages**

**(A-B)** Representative images of GAD65 immunofluorescence staining in the hippocampal CA1 region of WT and PPT1-KI mice at 3-4 months old **(A)** and 6-7 months old **(B)**. Scale bar: 50 µm.

**(C-D)** Western blot analysis of GAD65 expression of hippocampus of WT and PPT1-KI mice at 3-4 months old **(C)** and 6-7 months old **(D)**. Data are presented as mean ± SEM. T-test, **P* < 0.05; ns: no significant difference; N = 3 per group.


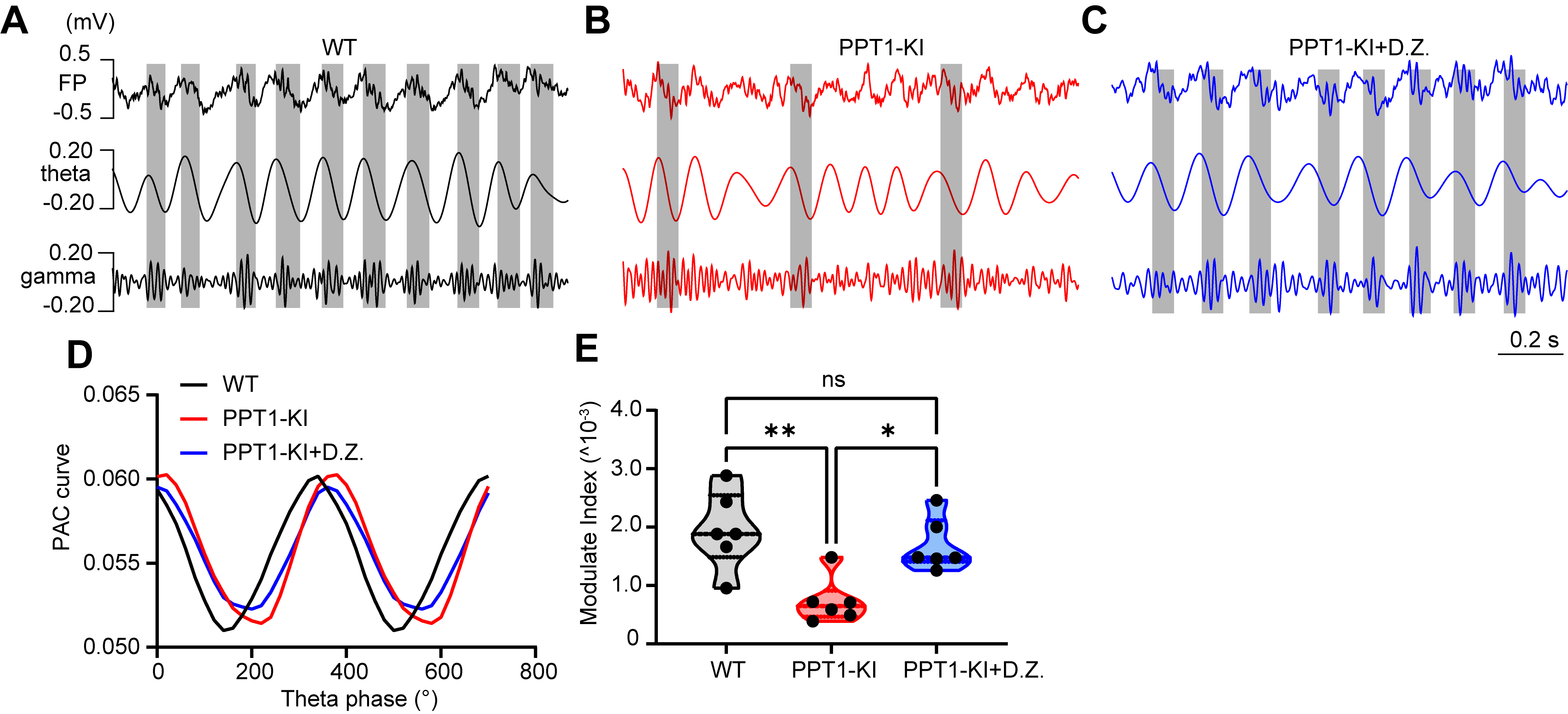


**Fig. S4 PPT1 deficiency impairs APC between theta and gamma oscillation**

**(A-C)** LFP signals and filtered theta (4~12 Hz) and gamma (30~80 Hz) recorded at CA1 region from WT **(A)**, PPT1-KI **(B)** mice, and PPT1-KI treated with D.Z. **(C)**.

**(D, E)** Comparison of the mean gamma amplitude per theta phase **(D)** and averaged MI **(E)**. Black: WT; red: PPT1-KI; blue: PPT1-KI treated with D.Z.. One-way ANOVA, **P* < 0.05; ***P* < 0.01. N = 6 mice for each group.


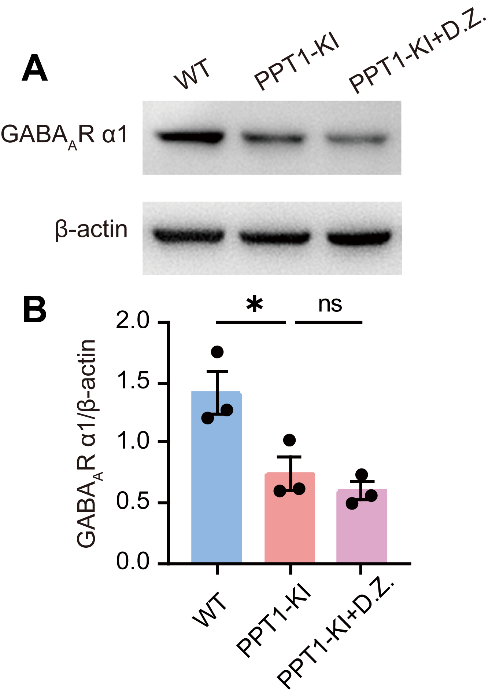


**Fig. S5 GABA_A_R α1 expression in 6- to 7-month-old mice after DZ treatment**

**(A)** Western blot analysis of GABA_A_R α1 expression in WT, PPT1-KI, and PPT1-KI mice treated with D.Z.. (6-7 months old).

**(B)** Bar chart showing GABA_A_R α1 expression levels. One-way ANOVA, **P* < 0.05; ns: no significant difference. N = 3 per each group.
